# Three-Component Decomposition Based on Stokes Vector for Compact Polarimetric SAR

**DOI:** 10.3390/s150924087

**Published:** 2015-09-18

**Authors:** Hanning Wang, Zhimin Zhou, John Turnbull, Qian Song, Feng Qi

**Affiliations:** 1 School of Electronic Science and Engineering, National University of Defense Technology, Changsha 410073, China; E-Mails: kdzhouzm@sina.cn (Z.Z.); danosong@gmail.com (Q.S.); 2 Information Technology Services, University of Birmingham, Birmingham B152TT, UK; E-Mail: johnturnbull2@googlemail.com; 3 Key Laboratory of Opt-Electronic Information Processing, Shenyang Institute of Automation, Chinese Academy of Science, Shenyang 110015, China; E-Mail: qifeng@riken.jp

**Keywords:** compact polarimetric SAR, Stokes vector, three-component decomposition, CTLR, DCP

## Abstract

In this paper, a three-component decomposition algorithm is proposed for processing compact polarimetric SAR images. By using the correspondence between the covariance matrix and the Stokes vector, three-component scattering models for CTLR and DCP modes are established. The explicit expression of decomposition results is then derived by setting the contribution of volume scattering as a free parameter. The degree of depolarization is taken as the upper bound of the free parameter, for the constraint that the weighting factor of each scattering component should be nonnegative. Several methods are investigated to estimate the free parameter suitable for decomposition. The feasibility of this algorithm is validated by AIRSAR data over San Francisco and RADARSAT-2 data over Flevoland.

## 1. Introduction

Compact polarimetric (CP) synthetic aperture radar (SAR) has been widely investigated over recent years. Compared with fully polarimetric (FP) SAR, CP SAR transmits only one polarization, thus avoiding the problems caused by high pulse repetition frequency (PRF), such as a low swath coverage, high data storage requirement and complicated system design. Several investigations demonstrate that CP SAR have the potential for a variety of remote sensing applications, such as soil moisture measurement [1], ship detection, oil spill identification [2,3], and vegetation height estimation [4].

According to the combination of polarization states, three typical CP SAR modes have been proposed, namely: π/4, circular transmission while linear reception (CTLR) and dual circular polarization (DCP). As the quantity of polarimetric information acquired by CP SAR is only half that of FP SAR, CP research has focused mainly on the extraction of scattering characterizations with similar finesse to that derived from FP systems [5]. 

Decomposition is an effective way to analyze the scattering data from a target. For CP SAR, several widely used decomposition methods have been proposed and improved, such as pseudo FP construction [6,7,8] and CP entropy/alpha decomposition [9,10,11]. In the paper [12] of Rui Guo *et al.*, in 2014, a three-component decomposition for a CP configuration is derived from a series of algebraic calculations, without reconstructing the pseudo FP information. Another school of thought is based on Stokes vector (SV), which is completely constructed from CP data. By using the polarization degree and relative phase calculated from SV, Raney *et al.* proposed the m−δ decomposition [13,14]. Cloude extended this idea to a compact decomposition theory in SV form [15]. 

In this paper, we focus on a three-component decomposition based on SV under the CTLR and DCP modes. We first establish the three-component model from the relationship between the covariance matrix and SV. To solve the underdetermined equations for CP decomposition, the contribution of volume scattering is taken as a free parameter, thus giving a complete set of solutions in an explicit format. According to the constraint that all weighting factors should be nonnegative, the depolarization degree is taken as the upper bound of volume scattering contribution. To validate the effectiveness of this algorithm, San Francisco data from AIRSAR and Flevoland data from RADARSAT-2 are used for testing.

Section 2 introduces the three-component model of SV. Section 3 and Section 4 present the deduction of decomposition for CTLR mode and DCP mode respectively. Section 5 compares this algorithm with Cloude CP and m−δ decompositions. Section 6 discusses methods to estimate the volume scattering contribution. Section 7 demonstrates the decomposition performance using real remote sensing data. Conclusions and future work are drawn in section 8.

## 2. Three-Component Model

Three stages are taken in turn to relate SV to decomposition theory: to begin with, we establish a three-component model of FP covariance matrix; this model is then transformed into the coherency matrix; finally the model is expressed by the SV under the CTLR and DCP modes respectively.

### 2.1. Three-Component Model of FP Covariance Matrix

The scattering characteristics of polarimetric SAR images can be evaluated by the second-order statistics of scattering matrix. Here we firstly focus on the covariance matrix

(1) $$C^{FP}=\langle k_{L}k_{L}^{H}\rangle$$

where 〈 〉 stands for the ensemble average in the data processing, ${\left(\;\right)}^{H}$ means transposition and conjugation, and subscript *L* states for lexicographic scattering vector. For monostatic FP SAR, the scattering vector $k_{L}$ is defined as [16]

(2) $$k_{L}={\left[\begin{array}{ccc} S_{HH} & \sqrt{2}S_{HV} & S_{VV} \end{array}\right]}^{T}$$

In three-component decomposition theory, the covariance matrix is modeled as the contribution of three scattering mechanisms: volume, double-bounce, and surface scatterings. According to [17], the three-component model of FP covariance matrix is given by

(3) $$C^{FP}=f_{v}\left[\begin{array}{ccc} 1 & 0 & 1/3\\ 0 & 2/3 & 0\\ 1/3 & 0 & 1 \end{array}\right]+f_{d}\left[\begin{array}{ccc} {\left|\alpha \right|}^{2} & 0 & \alpha \\ 0 & 0 & 0\\ \alpha^{*} & 0 & 1 \end{array}\right]+f_{s}\left[\begin{array}{ccc} {\left|\beta \right|}^{2} & 0 & \beta \\ 0 & 0 & 0\\ \beta^{*} & 0 & 1 \end{array}\right]$$

where $f_{v}$ , $f_{d}$ and $f_{s}$ are weighting factors of each component. 

### 2.2. Transforming Covariance Matrix to Coherency Matrix

The coherency matrix of monostatic FP SAR is based on the Pauli vector

(4) $$T=\langle k_{p}k_{p}^{H}\rangle$$

where the Pauli vector is defined as

(5) $$k_{p}=\frac{1}{\sqrt{2}}{\left[S_{HH}+S_{VV}\;S_{HH}-S_{VV}\;2S_{HV}\right]}^{T}$$

The relation between the covariance matrix and the coherency matrix is then derived from Equations (2) and (5)

(6) $$T=UC^{FP}U^{T}$$

where

(7) $$U=\frac{1}{\sqrt{2}}\left[\begin{array}{ccc} 1 & 0 & 1\\ 1 & 0 & -1\\ 0 & \sqrt{2} & 0 \end{array}\right]$$

According to Equations (3) and (6), we obtain the three-component model of the coherency matrix

(8) $$\begin{array}{c} T=\frac{2}{3}f_{v}\left[\begin{array}{ccc} 2 & 0 & 0\\ 0 & 1 & 0\\ 0 & 0 & 1 \end{array}\right]+\frac{1}{2}f_{d}\left[\begin{array}{ccc} |\alpha {|}^{2}+\alpha +\alpha^{*}+1 & |\alpha {|}^{2}-\alpha +\alpha^{*}-1 & 0\\ |\alpha {|}^{2}+\alpha -\alpha^{*}-1 & |\alpha {|}^{2}-\alpha -\alpha^{*}+1 & 0\\ 0 & 0 & 0 \end{array}\right]\\ +\frac{1}{2}f_{s}\left[\begin{array}{ccc} |\beta {|}^{2}+\beta +\beta^{*}+1 & |\beta {|}^{2}-\beta +\beta^{*}-1 & 0\\ |\beta {|}^{2}+\beta -\beta^{*}-1 & |\alpha {|}^{2}-\beta -\beta^{*}+1 & 0\\ 0 & 0 & 0 \end{array}\right] \end{array}$$

### 2.3. Mapping Coherency Matrix to Output SV under CTLR Mode

For CTLR mode, assuming the transmitted polarization is right hand circular, the normalized polarization of the transmitted wave is [16]

(9) $$E_{t}^{CTLR}=\frac{1}{\sqrt{2}}\left[\begin{array}{c} 1\\ -j \end{array}\right]$$

Correspondingly, the SV of the transmitted wave is given by

(10) $$g_{t}^{CTLR}={\left[\begin{array}{cccc} 1 & 0 & 0 & -1 \end{array}\right]}^{T}$$

The SV of the scattered wave is related to that of the incident wave by Mueller matrix

(11) $$g_{s}^{CTLR}={\mathrm{Mg}}_{t}^{CTLR}$$

The Mueller matrix can be expressed by Huynen parameters as [18,19]

(12) $$M=\left[\begin{array}{cccc} A_{0}+B_{0} & C & H & F\\ C & A_{0}+B & E & G\\ H & E & A_{0}-B & D\\ -F & -G & -D & A_{0}-B_{0} \end{array}\right]$$

Therefore, the SV of the scattered wave is written as

(13) $$g_{s}^{CTLR}=\left[\begin{array}{c} g_{0}\\ g_{1}\\ g_{2}\\ g_{3} \end{array}\right]=\left[\begin{array}{c} A_{0}+B_{0}-F\\ C-G\\ H-D\\ -A_{0}+B_{0}-F \end{array}\right]$$

Notice that the coherency matrix can also be expressed by Huynen parameters as [18,19]

(14) $$T=\left[\begin{array}{ccc} t_{11} & t_{12} & t_{13}\\ t_{12}^{*} & t_{22} & t_{23}\\ t_{13}^{*} & t_{23}^{*} & t_{33} \end{array}\right]=\left[\begin{array}{ccc} 2A_{0} & C-jD & H+jG\\ C+jD & B_{0}+B & E+jF\\ H-jG & E-jF & B_{0}-B \end{array}\right]$$

Using Equations (13) and (14), the SV of the scattered wave is related to the coherency matrix

(15) gsCTLR=[g0g1g2g3]=[12(t11+t22+t33)−Im(t23)Re(t12)−Im(t13)Re(t13)+Im(t12)12(−t11+t22+t33)−Im(t23)]

The three-component model based on SV under the CTLR mode is derived from Equations (8) and (15) as

(16) $$g_{s}^{CTLR}=\left[\begin{array}{c} g_{0}\\ g_{1}\\ g_{2}\\ g_{3} \end{array}\right]=f_{v}\left[\begin{array}{c} 2\\ 0\\ 0\\ 0 \end{array}\right]+f_{d}\left[\begin{array}{c} |\alpha {|}^{2}+1\\ |\alpha {|}^{2}-1\\ j(\alpha -\alpha^{*})\\ -\alpha -\alpha^{*} \end{array}\right]+f_{s}\left[\begin{array}{c} |\beta {|}^{2}+1\\ |\beta {|}^{2}-1\\ j(\beta -\beta^{*})\\ -\beta -\beta^{*} \end{array}\right]$$

### 2.4. Mapping Coherency Matrix to Output SV under DCP Mode

For the DCP mode, we also assume the transmitted polarization as the right hand circular. The scattering vector in this case is

(17) $$E_{s}^{DCP}=\left[\begin{array}{c} E_{RR}\\ E_{LR} \end{array}\right]=\left[\begin{array}{cc} 1/\sqrt{2} & -j/\sqrt{2}\\ -j/\sqrt{2} & 1/\sqrt{2} \end{array}\right]\left[\begin{array}{cc} S_{HH} & S_{HV}\\ S_{VH} & S_{VV} \end{array}\right]\left[\begin{array}{c} 1/\sqrt{2}\\ -j/\sqrt{2} \end{array}\right]$$

We can now obtain the relationship between the scattering vector under the DCP mode and that under the CTLR mode

(18) $$E_{s}^{DCP}=\left[\begin{array}{c} E_{RR}\\ E_{LR} \end{array}\right]=\left[\begin{array}{cc} 1/\sqrt{2} & -j/\sqrt{2}\\ -j/\sqrt{2} & 1/\sqrt{2} \end{array}\right]\left[\begin{array}{c} E_{HR}\\ E_{VR} \end{array}\right]$$

In this paper, we define the SV of the scattered wave under the DCP mode as

(19) $$g_{s0}^{DCP}=\;<|E_{RR}{|}^{2}+|E_{LR}{|}^{2}>\;$$

(20) $$g_{s1}^{DCP}=\;<|E_{RR}{|}^{2}-|E_{LR}{|}^{2}>\;$$

(21) gs2DCP=2Re<ERRELR∗>

(22) gs3DCP=−2Im<ERRELR∗> 

For simplicity of expression, the SV elements under DCP mode are also rewritten as $g_{0}$, $g_{1}$, $g_{2}$ and $g_{3}$. From Equation (18), the following relationships are obtained as

(23) $$g_{0}=g_{s0}^{DCP}=\;<|E_{HR}{|}^{2}+|E_{VR}{|}^{2}>\;=g_{s0}^{CTLR}$$

(24) g1=gs1DCP= −2Im<EHREVR∗> =gs3CTLR

(25) g2=gs2DCP=2Re<EHREVR∗> =gs2CTLR

(26) $$g_{3}=g_{s3}^{DCP}=-<|E_{HR}{|}^{2}+|E_{VR}{|}^{2}>\;=-g_{s1}^{CTLR}$$

Therefore, by exchanging the first and third elements in the SV under the CTLR mode, we derive the SV under the DCP mode

(27) gsDCP=[A0+B0−F−A0+B0−FH−DG−C]=[12(t11+t22+t33)−Im(t23)12(−t11+t22+t33)−Im(t23)Re(t13)+Im(t12)−Re(t12)+Im(t13)]

The three-component model based on SV under the DCP mode is derived as

(28) $$g_{s}^{DCP}=\left[\begin{array}{c} g_{0}\\ g_{1}\\ g_{2}\\ g_{3} \end{array}\right]=f_{v}\left[\begin{array}{c} 2\\ 0\\ 0\\ 0 \end{array}\right]+f_{d}\left[\begin{array}{c} |\alpha {|}^{2}+1\\ -\alpha -\alpha^{*}\\ j(\alpha -\alpha^{*})\\ -{\left|\alpha \right|}^{2}+1 \end{array}\right]+f_{s}\left[\begin{array}{c} |\beta {|}^{2}+1\\ -\beta -\beta^{*}\\ j(\beta -\beta^{*})\\ -|\beta {|}^{2}+1 \end{array}\right]$$

## 3. Explicit Expressions of Three-component Decomposition for CTLR Mode

Essentially, the three-component decomposition of SV for CP SAR implies solving underdetermined equations. From Equation (16), there are only four constraint equations, while the number of unknowns is seven. According to Freeman and Durden’s algorithm [17], the number of unknowns can be reduced to five by setting α=−1 or β=1, however, one free parameter still remains. In this paper, we take the volume scattering component as the free parameter.

Setting $x=2f_{v}$ and substituting it into Equation (16), we obtain the Rest Scattering Model (RSM) with double-bounce and surface scattering components as

(29) $$\left[\begin{array}{c} g_{0}-x\\ g_{1}\\ g_{2}\\ g_{3} \end{array}\right]=f_{d}\left[\begin{array}{c} |\alpha {|}^{2}+1\\ |\alpha {|}^{2}-1\\ j(\alpha -\alpha^{*})\\ -\alpha -\alpha^{*} \end{array}\right]+f_{s}\left[\begin{array}{c} |\beta {|}^{2}+1\\ |\beta {|}^{2}-1\\ j(\beta -\beta^{*})\\ -\beta -\beta^{*} \end{array}\right]$$

According to Freeman and Durden’s algorithm, we fix α=−1 or β=1 according to the sign of Re(fdα+fsβ). From Equation (29), $f_{d}\alpha +f_{s}\beta$ is obtained as

(30) $$\alpha f_{d}+\beta f_{s}=-0.5\left(g_{3}+jg_{2}\right)$$

Because both $g_{2}$ and $g_{3}$ are real, we have

(31) sign[Re(fdα+fsβ)]=sign(−g3)

### 3.1. Calculation of Unknowns When $g_{3}<0$

According to the discussion above, α is fixed as −1 when $g_{3}<0$ , and Equation (29) becomes

(32) $$\left[\begin{array}{c} g_{0}-x\\ g_{1}\\ g_{2}\\ g_{3} \end{array}\right]=f_{d}\left[\begin{array}{c} 2\\ 0\\ 0\\ 2 \end{array}\right]+f_{S}\left[\begin{array}{c} |\beta {|}^{2}+1\\ |\beta {|}^{2}-1\\ j(\beta -\beta^{*})\\ -\beta -\beta^{*} \end{array}\right]$$

To eliminate $f_{d}$ and $f_{s}$, take the ratio to give

(33) $$\frac{g_{1}-jg_{2}}{g_{0}-g_{3}-x}=\frac{\beta -1}{\beta +1}$$

Thus β is given by

(34) $$\beta =\frac{g_{1}+g_{0}-g_{3}-x-jg_{2}}{-g_{1}+g_{0}-g_{3}-x+jg_{2}}$$

$f_{d}$ and $f_{s}$ are derived by substituting Equation (34) into Equation (32)

(35) $$f_{d}=\frac{(g_{0}+g_{3}-x)(g_{0}-g_{3}-x)-g_{1}^{2}-g_{2}^{2}}{4(g_{0}-g_{3}-x)}$$

(36) $$f_{s}=\frac{{\left(g_{1}-g_{0}+g_{3}+x\right)}^{2}+g_{2}^{2}}{4(g_{0}-g_{3}-x)}$$

Now we concern the value range of *x*. With the constraint that all weighting factors are nonnegative, *x* must satisfy the following inequalities

(37) $$\{\begin{array}{c} 0\leq x\leq g_{0}-g_{3}\\ (g_{0}+g_{3}-x)(g_{0}-g_{3}-x)-g_{1}^{2}-g_{2}^{2}\geq 0 \end{array}$$

The second inequality in Equation (37) is quadratic

(38) $$x^{2}-2g_{0}x+(g_{0}^{2}-g_{1}^{2}-g_{2}^{2}-g_{3}^{2})\geq 0$$

and the corresponding value range for *x* is

(39) $$\left(-\infty ,x_{1})\cup (x_{2},\infty \right)$$

where

(40) $$x_{1}=g_{0}-\sqrt{g_{1}^{2}+g_{2}^{2}+g_{3}^{2}}$$

(41) $$x_{2}=g_{0}+\sqrt{g_{1}^{2}+g_{2}^{2}+g_{3}^{2}}$$

Because *x* cannot be larger than $g_{0}-g_{3}$ according to the first inequality in Equation (37), the value in $\left(x_{2},\infty \right)$ is not acceptable. Based on the analysis above, the value range of *x* is given by

(42) $$x\in [0,x_{\max}]$$

(43) $$x_{\max}=Min\left\{g_{0}-g_{3},x_{1}\right\}$$

It is easy to show that $x_{1}\leq g_{0}-g_{3}$, and thus Equation (42) is rewritten as

(44) $$x\in [0,x_{1}]$$

The contribution of each scattering mechanism is estimated with elements of the SV

(45) $$P_{v}=x$$

(46) $$P_{d}=f_{d}(|\alpha {|}^{2}+1)=\frac{(g_{0}+g_{3}-x)(g_{0}-g_{3}-x)-g_{1}^{2}-g_{2}^{2}}{2(g_{0}-g_{3}-x)}$$

(47) $$P_{s}=f_{s}(|\beta {|}^{2}+1)=\frac{{\left(g_{0}-g_{3}-x\right)}^{2}+g_{1}^{2}+g_{2}^{2}}{2(g_{0}-g_{3}-x)}$$

It is easy to verify

(48) $$P_{v}+P_{d}+P_{s}=g_{0}$$

### 3.2. Calculation of Unknowns When $g_{3}>0$

When $g_{3}>0$, β is fixed as 1, and Equation (16) becomes

(49) $$\left[\begin{array}{c} g_{0}-x\\ g_{1}\\ g_{2}\\ g_{3} \end{array}\right]=f_{d}\left[\begin{array}{c} |\alpha {|}^{2}+1\\ |\alpha {|}^{2}-1\\ j(\alpha -\alpha^{*})\\ -\alpha -\alpha^{*} \end{array}\right]+f_{S}\left[\begin{array}{c} 2\\ 0\\ 0\\ -2 \end{array}\right]$$

With a similar method, contributions of each scattering mechanism are obtained as

(50) $$P_{v}=x$$

(51) $$P_{d}=f_{d}(|\alpha {|}^{2}+1)=\frac{{\left(g_{0}+g_{3}-x\right)}^{2}+g_{1}^{2}+g_{2}^{2}}{2(g_{0}+g_{3}-x)}$$

(52) $$P_{s}=f_{s}(|\beta {|}^{2}+1)=\frac{(g_{0}-g_{3}-x)(g_{0}+g_{3}-x)-g_{1}^{2}-g_{2}^{2}}{2(g_{0}+g_{3}-x)}$$

where

(53) $$x\in [0,x_{1}]$$

It is interesting to notice that

(54) $$x_{1}=g_{0}\left(1-m\right)$$

where $m=\frac{\sqrt{g_{1}^{2}+g_{2}^{2}+g_{3}^{2}}}{g_{0}}$ denotes the polarization degree. 

## 4. Explicit Expressions of Three-component Decomposition for DCP Mode

Setting $x=2f_{v}$ and substituting into Equation (28), we obtain the RSM with double-bounce and surface scattering components as

(55) $$\left[\begin{array}{c} g_{0}-x\\ g_{1}\\ g_{2}\\ g_{3} \end{array}\right]=f_{d}\left[\begin{array}{c} |\alpha {|}^{2}+1\\ -\alpha -\alpha^{*}\\ j(\alpha -\alpha^{*})\\ -|\alpha {|}^{2}+1 \end{array}\right]+f_{s}\left[\begin{array}{c} |\beta {|}^{2}+1\\ -\beta -\beta^{*}\\ j(\beta -\beta^{*})\\ -|\beta {|}^{2}+1 \end{array}\right]$$

Based on Freeman and Durden’s assumption, we fix α=−1 or β=1 according to the sign of Re(fdα+fsβ). From (55), we have

(56) sign[Re(fdα+fsβ)]=sign(−g1)

### 4.1. Calculation of Unknowns When $g_{1}<0$

As in the discussion above, α is fixed as −1 when $g_{1}<0$. Using a method similar to that developed in Section 3, we derive contributions of each scattering mechanism

(57) $$P_{v}=x$$

(58) $$P_{d}=\frac{(g_{0}+g_{1}-x)(g_{0}-g_{1}-x)-g_{3}^{2}-g_{2}^{2}}{2(g_{0}-g_{1}-x)}$$

(59) $$P_{s}=\frac{{\left(g_{0}-g_{1}-x\right)}^{2}+g_{3}^{2}+g_{2}^{2}}{2(g_{0}-g_{1}-x)}$$

where

(60) $$x\in [0,x_{1}]$$

### 4.2. Calculation of Unknowns When $g_{1}>0$

When $g_{1}>0$, β is fixed as 1, and contributions of each scattering mechanism are obtained as

(61) $$P_{v}=x$$

(62) $$P_{d}=\frac{{\left(g_{0}+g_{1}-x\right)}^{2}+g_{3}^{2}+g_{2}^{2}}{2(g_{0}+g_{1}-x)}$$

(63) $$P_{s}=\frac{(g_{0}-g_{1}-x)(g_{0}+g_{1}-x)-g_{3}^{2}-g_{2}^{2}}{2(g_{0}+g_{1}-x)}$$

where

(64) $$x\in [0,x_{1}]$$

## 5. Comparison with Cloude CP and m−δ Decompositions

This section compares the proposed algorithm with other two SV based decompositions. For simplicity, only the CTLR mode is taken for analysis.

### 5.1. Cloude CP Decomposition

According to [15], for a general rank-1 symmetric scattering mechanism, the SV of the scattering wave under the CTLR mode can be written as

(65) $$g_{s}^{CTLR}=\frac{m_{s}}{2}{\left[\begin{array}{cccc} 1 & \sin2\alpha_{s}\cos\varphi & \sin2\alpha_{s}\sin\varphi & -\cos2\alpha_{s} \end{array}\right]}^{T}$$

where $\alpha_{s}$ and φ are scattering parameters, which can be estimated by the SV elements

(66) $$\alpha_{s}=\frac{1}{2}\tan^{-1}\left(\frac{\sqrt{g_{1}^{2}+g_{2}^{2}}}{-g_{3}}\right)$$

(67) $$\varphi =\tan^{-1}\frac{g_{2}}{g_{1}}$$

The decomposition proposed by Cloude *etc.* is given as following equations

(68) $$P_{v}=g_{0}(1-m)=g_{0}-\sqrt{g_{1}^{2}+g_{2}^{2}+g_{3}^{2}}$$

(69) $$P_{d}=\frac{1}{2}g_{0}m(1-\cos2\alpha_{s})=\frac{1}{2}(\sqrt{g_{1}^{2}+g_{2}^{2}+g_{3}^{2}}+g_{3})$$

(70) $$P_{s}=\frac{1}{2}g_{0}m(1+\cos2\alpha_{s})=\frac{1}{2}(\sqrt{g_{1}^{2}+g_{2}^{2}+g_{3}^{2}}-g_{3})$$

Here the depolarized component is regarded as the contribution of volume scattering. $P_{d}$ and $P_{s}$ are estimated from the polarized component and $g_{3}$.

### 5.2. m−δ Decomposition 

In the m−δ decomposition [13,14], the contribution of volume scattering is also estimated as the depolarized component, while the relative phase δ is taken as the factor to split the polarized component into $P_{d}$ and $P_{s}$

(71) $$\delta =\tan^{-1}\left(\frac{g_{3}}{g_{2}}\right)$$

(72) $$P_{v}=g_{0}(1-m)=g_{0}-\sqrt{g_{1}^{2}+g_{2}^{2}+g_{3}^{2}}$$

(73) $$P_{d}=\frac{1}{2}g_{0}m(1+\sin\delta )=\frac{1}{2}\left(\sqrt{g_{1}^{2}+g_{2}^{2}+g_{3}^{2}}+g_{3}\frac{\sqrt{g_{1}^{2}+g_{2}^{2}+g_{3}^{2}}}{\sqrt{g_{2}^{2}+g_{3}^{2}}}\right)$$

(74) $$P_{s}=\frac{1}{2}g_{0}m(1-\sin\delta )=\frac{1}{2}\left(\sqrt{g_{1}^{2}+g_{2}^{2}+g_{3}^{2}}-g_{3}\frac{\sqrt{g_{1}^{2}+g_{2}^{2}+g_{3}^{2}}}{\sqrt{g_{2}^{2}+g_{3}^{2}}}\right)$$

### 5.3. Difference Analysis

Different from the above two methods, the decomposition proposed in this paper takes the depolarized component as the upper bound of volume scattering contribution. Three conclusions are obtained from Equations (44)–(47) and (50)–(54):

(a) In our case, we consider volume scattering can be less than the depolarized component that [13,14,15] used
(b) Besides the volume scattering, the combined effect of double-bounce and surface scatterings also contributes to depolarization
(c) When the depolarized component is only caused by volume scattering *i.e.*, *x* = *x*_1_ this algorithm degrades to a two-component decomposition.

## 6. Value Estimation of *x*

The difficult part of our algorithm is to estimate the value of the unknown parameter *x*. Based on the analysis above, three preliminary methods for value estimation are proposed:

(a) Assuming the depolarization is only caused by the volume scattering
  (75) $$x=x_{1}=g_{0}-\sqrt{g_{1}^{2}+g_{2}^{2}+g_{3}^{2}}$$

As mentioned in Section 5.3, one of $P_{d}$ and $P_{s}$ would be null in this case. Thus the decomposition now becomes two components (volume scattering and ground scattering), in which the ground scattering component switches between double-bounce and surface scattering.

(b) Considering the fact that the combined effect of double-bounce and surface scatterings also contributes to depolarization, we define

(76) $$x=px_{1}=p\cdot Span\left(1-m\right),\;0\leq p\leq 1$$

where *p* is defined as the volume scattering factor. Method (a) is the special case when *p* = 1, while the suitable value of *p* should be selected according to the quality of decomposition. In the next section, two typical polarimetric SAR data sets are taken to analyze the decomposition quality under different values of *p*. The result shows that some pixels in urban areas also have a considerable depolarized component, and they can be well distinguished from vegetation areas when 0.5≤p≤0.8. It indicates that the volume scattering component contributes to the main part of depolarization, but not all. Since this interval is wide and the performance is robust, it seems that p∈[0.5,0.8] could be suggested for other CP data.

(c) Reconstructing $\langle {\left|S_{HV}\right|}^{2}\rangle$ from CP data, the value of *x* is then derived as
  (77) $$x=\min\left\{4\langle {\left|S_{HV}\right|}^{2}\rangle ,\;x_{1}\right\}$$

To satisfy the nonnegative requirement, here we use the depolarized component as the threshold to curb the overestimation of volume scattering contribution. The precision of reconstruction depends on the coincidence rate of remote sensing data to the reflection symmetrical hypothesis and empirical formulas. For the reconstruction algorithm proposed by Souyris [6] and Nord [7], the empirical formula is given by

(78) $$\frac{<|S_{HV}{|}^{2}>}{<|S_{HH}{|}^{2}>+<|S_{VV}{|}^{2}>}=\frac{1-|r|}{N}$$

where

(79) $$r=\frac{<S_{HH}S_{VV}^{*}>}{\sqrt{<|S_{HH}{|}^{2}><|S_{VV}{|}^{2}>}}$$

The value of *N* is 4 in Souyris’ formula. Differently, Nord estimated *N* as

(80) $$N=\frac{|<S_{HH}-S_{VV}>{|}^{2}}{<|S_{HV}{|}^{2}>|}$$

Souyris’ empirical formula is suitable to the model of volume scattering, however it does not fit the other two scattering models. To avoid this problem, the formula is modified as

(81) $$\frac{<|S_{HV}{|}^{2}>}{\left(<|S_{HH}{|}^{2}>+|S_{VV}{|}^{2}>+2<|S_{HV}{|}^{2}>\right)}=w_{v}\frac{1-|r|}{16/3}$$

where $w_{v}$ is the weighting of volume scattering component

(82) $$w_{v}=\frac{P_{v}}{P_{v}+P_{d}+P_{s}}$$

Correspondingly, the estimation of $<|S_{HV}{|}^{2}>$ becomes

(83) $$<|S_{HV}{|}^{2}>=w_{v}\frac{1-|r|}{8/3}g_{0}$$

Based on the analysis above, the major steps to reconstruct $<|S_{HV}{|}^{2}>$ are given as follows: Denoting $X=<|S_{HV}{|}^{2}>$, the upper bound of depolarized component is taken as the initial condition

(84) $$X_{\left(0\right)}=x_{1}/4,\;x_{\left(0\right)}=x_{1}$$

$P_{v(0)}$, $P_{d(0)}$, $P_{s(0)}$ and $w_{v(0)}$ are obtained from the decomposition algorithm given in Section 3 and Section 4. Then a recursive process is set up as:

(85) $$r_{\left(k+1\right)}=\frac{-(g_{3}+jg_{2})+X_{\left(k\right)}}{\sqrt{\left(g_{0}+g_{1}-X_{\left(k\right)})(g_{0}-g_{1}-X_{\left(k\right)}\right)}}$$

(86) $$X_{\left(k+1\right)}=w_{v(k)}\frac{1-|r_{\left(k+1\right)}|}{8/3}g_{0}$$

(87) $$x_{\left(k+1\right)}=\min\left\{4X_{\left(k+1\right)},x_{1}\right\}$$

$P_{v(k+1)}$, $P_{d(k+1)}$, $P_{s(k+1)}$ and $w_{v(k+1)}$ are derived from the decomposition algorithm, with $x_{\left(k+1\right)}$ as the volume scattering component.

## 7. Performance Demonstration

The feasibility of the proposed decomposition algorithm is tested with two data sets acquired by the NASA/JPL AIRSAR system and RADARSAT-2 respectively.

To analyze the performance of CP decomposition, FP decomposition results are taken as the standard. Since original Freeman–Durden decomposition in FP mode may cause negative components, an improved decomposition algorithm proposed in [20] is used to process FP data.

The quality of CP decomposition is measured by the classification conformity degree of CP mode compared to FP mode. Three types of statistics are considered for measurement:

(a) Conformity degree to FP mode for each class (CDC)
(b) Averaged conformity degree for the whole image (ADI)
(c) Proportion of each class in the image (PCI)

CDC and ADI are obtained from the classification confusion matrix. Taking the AIRSAR data over San Francisco for example, by comparing the classification conformity between decompositions under FP mode and CTLR mode when *p* = 0.65, the confusion matrix is shown in Table 1.

**Table 1:** Classification confusion matrix.

	Volume	Double	Surface
Volume	78.61%	20.37%	1.02%
Double	24.21%	75.76%	0.03%
Surface	8.75%	0.37%	90.89%

CDC consists of the diagonal elements (78.61%, 75.76% and 90.89%). Correspondingly, ADI is calculated as the averaged value of the three elements in CDC:

ADI=(78.61%+75.76%+90.89%)/3=81.75%

PCI indicates the quality of CP decomposition from another aspect, as shown in Table 2. 

**Table 2:** Comparison by Proportion of Each Class in the image (PCI).

Decomposition	PCI		
	Volume	Double	Surface
FP	22.30%	33.24%	44.46%
CTLR (*p* = 1)	51.33%	11.10%	37.57%
CTLR (*p* = 0.65)	29.47%	29.89%	40.64%

In comparison, the PCI when *p* = 0.65 is more similar to that under FP mode, which indicates a better decomposition performance than the case when *p* = 1.

Since ADI is a single value which is convenient to quantify the performance of CP decomposition, we take it as the major criterion for measurement. Besides that, CDC and PCI are also regarded as supplementary criteria. 

### 7.1. AIRSAR Data over San Francisco

**Figure 1:**
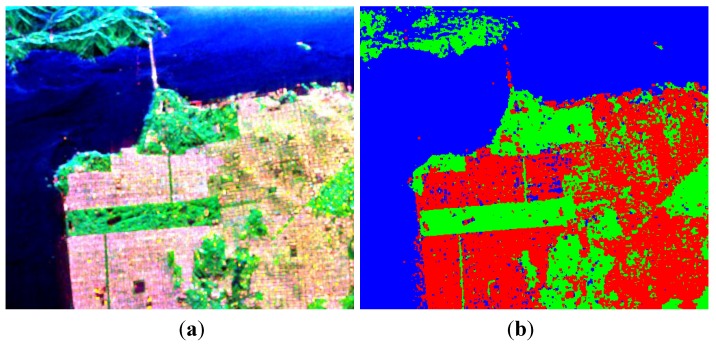
Three-component decomposition under fully polarimetric (FP) mode (the original data is smoothed by a 7 × 7 pixel window): (**a**) pseudo color image and (**b**) classification.

The image over San Francisco was acquired by AIRSAR at L-band, with the image size of 900 × 1024. This region contains three typical terrain types: vegetation areas, man-made structures and the sea. Figure 1a shows the pseudo color image of the decomposition result under the FP mode. 

In Figure 1a, the three colors red, green and blue correspond to $P_{d}$, $P_{v}$ and $P_{s}$ respectively. Classifying each pixel with the largest component, the results are shown in Figure 1b.

**Figure 2:**
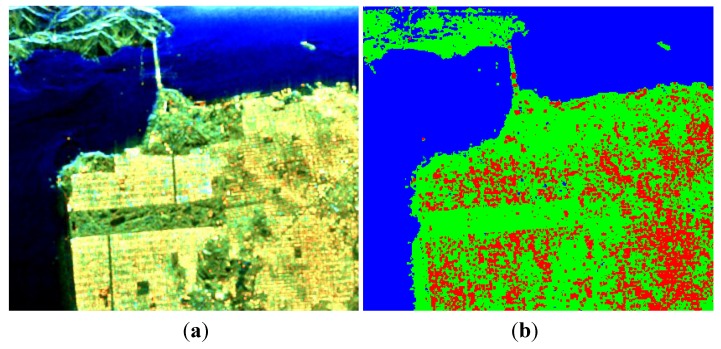
Three-component decomposition under circular transmission while linear reception (CTLR) mode when *p* = 1 (the original data is smoothed by a 7 × 7 pixel window): (**a**) pseudo color image and (**b**) classification.

The SVs for each pixel under the three CP modes are built from FP data, and then the algorithm described in Section 3 and Section 4 is adopted for decomposition. As mentioned in Section 6, the value estimation of volume scattering component is critical for our decomposition algorithm. To get an initial impression, we first assume that the depolarization is only caused by the volume scattering *i.e.*, *p* = 1 in Equation (76), the corresponding decomposition results are shown in Figure 2.

**Figure 3:**
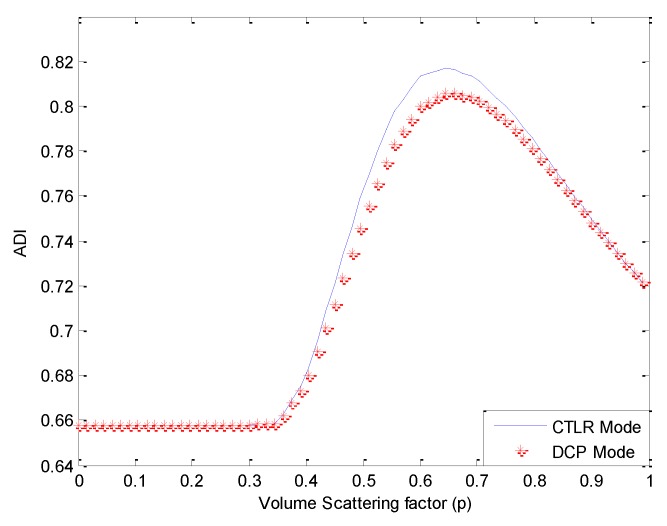
Averaged conformity degree for the whole image (ADI) under different values of *p.*

It is obvious to observe that a certain number of double-bounce scatters are misclassified as volume scatters when *p* = 1. In order to select a value of *x* suitable for decomposition, we calculate ADI under different values of *p*, as shown in Figure 3.

**Figure 4:**
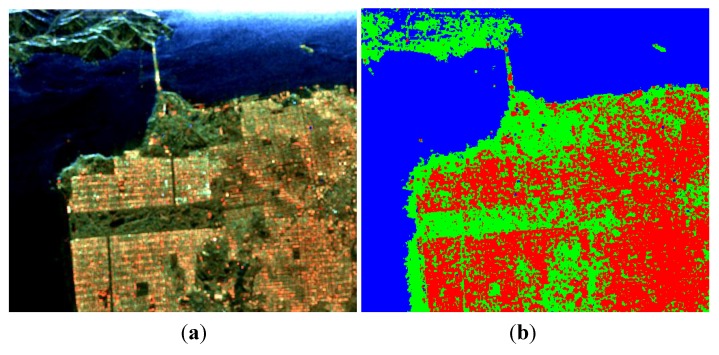
Three-component decomposition under CTLR mode when *p* = 0.65 (the original data is smoothed by a 7 × 7 pixel window): (**a**) pseudo color image and (**b**) classification.

Of the values for *p* tested, a satisfactory ADI can be obtained when 0.5≤p≤0.8, and a maximum is reached when *p* = 0.65, as shown in Figure 4. 

**Figure 5:**
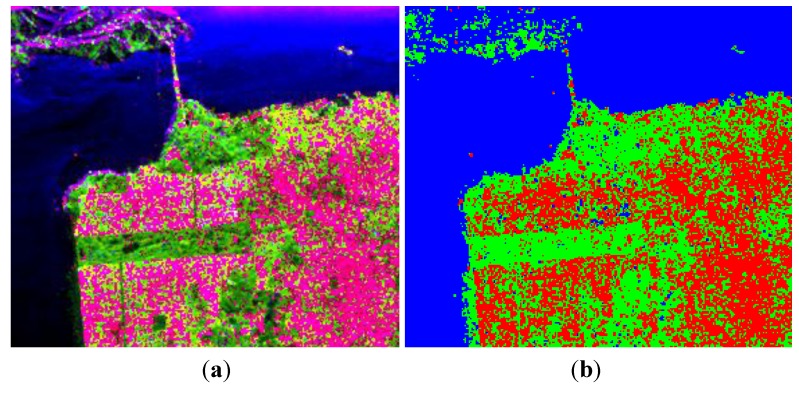
Three-component decomposition under CTLR mode after reconstruction (the original data is smoothed by a 7 × 7 pixel window): (**a**) pseudo color image and (**b**) classification.

Reconstructing $\langle {\left|S_{HV}\right|}^{2}\rangle$ with the algorithm proposed in Section 6, the corresponding decomposition results are given in Figure 5.

**Figure 6:**
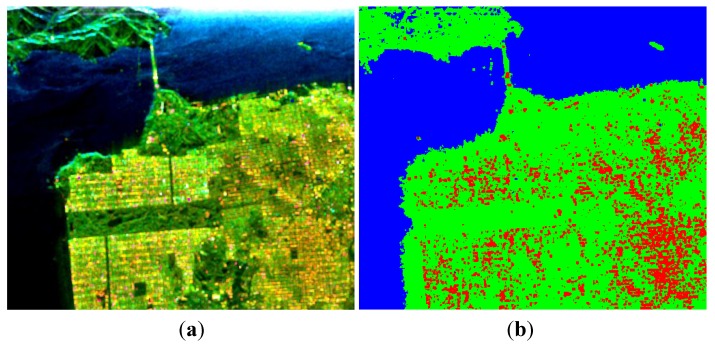
Three-component decomposition using Cloude compact polarimetric (CP) decomposition (the original data is smoothed by a 7 × 7 pixel window): (**a**) pseudo color image and (**b**) classification.

**Figure 7:**
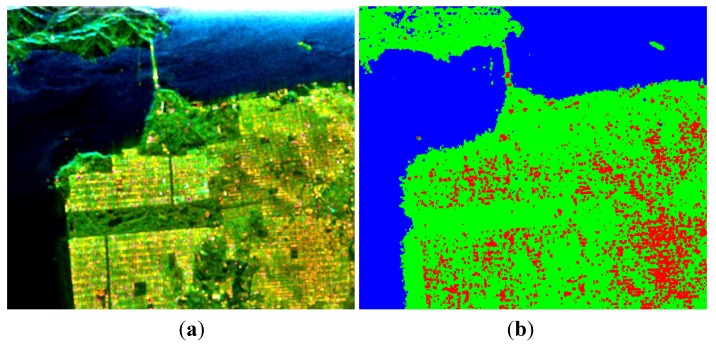
Three-component decomposition using m−δ decomposotion (the original data is smoothed by a 7 × 7 pixel window): (**a**) pseudo color image and (**b**) classification.

We also process the data with Cloude CP and m−δ decompositions, as shown in Figure 6 and Figure 7. The corresponding numerical comparisons are given in Table 3 and Table 4.

**Table 3:** Comparison by Conformity Degree to FP Mode for Each Class (CDC) and Averaged Conformity Degree for the Whole Image (ADI).

Decomposition	CDC			ADI
	Volume	Double	Surface	
CTLR (*p* = 1)	98.56%	32.33%	84.49%	71.79%
CTLR (*p* = 0.65)	78.61%	75.76%	90.89%	81.75%
CTLR(Reconstruction, us)	76.88%	68.26%	94.71%	79.95%
CTLR(Reconstruction, Nord)	57.94%	86.51%	87.88%	77.44%
CTLR (Cloude *etc.*)	99.03%	26.85%	83.48%	69.79%
CTLR (m−δ)	98.85%	29.03%	84.00%	70.63%
DCP (*p* = 1)	98.35%	31.66%	85.54%	71.85%
DCP (*p* = 0.65)	76.31%	74.43%	91.18%	80.64%
DCP(Reconstruction, us)	80.56%	64.82%	94.36%	79.91%
DCP (Reconstruction, Nord)	62.30%	84.56%	87.29%	78.05%

**Table 4:** Comparison by Proportion of each Class in the Image (PCI).

Decomposition	PCI		
	Volume	Double	Surface
FP	22.30%	33.24%	44.46%
CTLR (*p* = 1)	51.33%	11.10%	37.57%
CTLR (*p* = 0.65)	29.47%	29.89%	40.64%
CTLR(Reconstruction, us)	26.46%	29.86%	43.69%
CTLR(Reconstruction, Nord)	22.30%	38.07%	39.63%
CTLR (Cloude *etc.*)	53.74%	9.15%	37.11%
CTLR(m−δ)	52.73%	9.92%	37.35%
DCP (*p* = 1)	51.05%	10.90%	38.05%
DCP (*p* = 0.65)	29.30%	29.93%	40.86%
DCP(Reconstruction, us)	24.68%	32.00%	43.31%
DCP (Reconstruction, Nord)	24.33%	36.33%	39.34%

### 7.2. RADARSAT-2 Data over Flevoland

**Figure 8:**
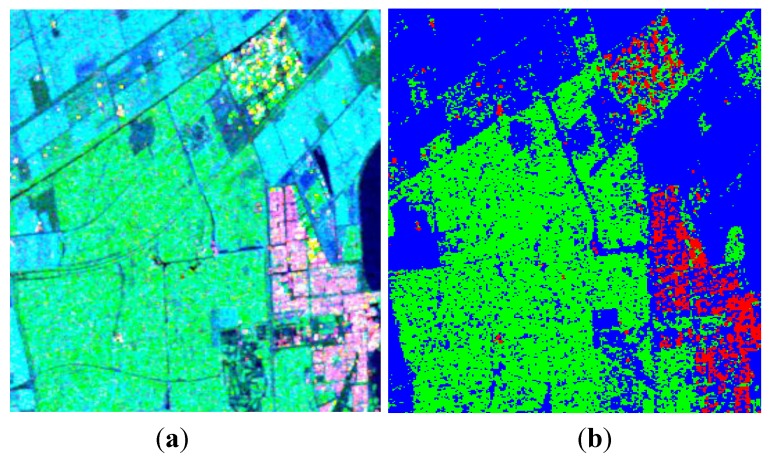
Three-component decomposition under FP mode (the original data is smoothed by a 7 × 7 pixel window): (**a**) pseudo color image and (**b**) classification.

The image over Flevoland was acquired by RADARSAT-2 at C-band, with the image size of 1513 × 1009. This region contains four major terrain types: forests, man-made structures, the lake and farms. Figure 8 shows the decomposition result under the FP mode by using the improved Freeman-Durden decomposition algorithm. 

**Figure 9:**
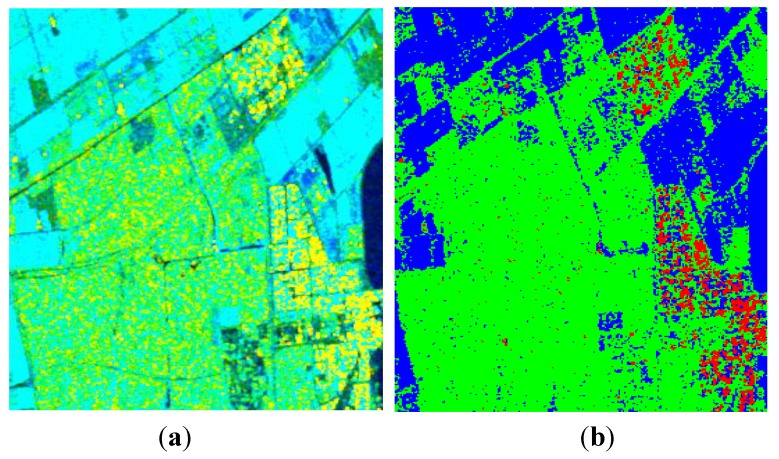
Three-component decomposition under FP mode when *p* = 1 (the original data is smoothed by a 7 × 7 pixel window): (**a**) pseudo color image and (**b**) classification.

**Figure 10:**
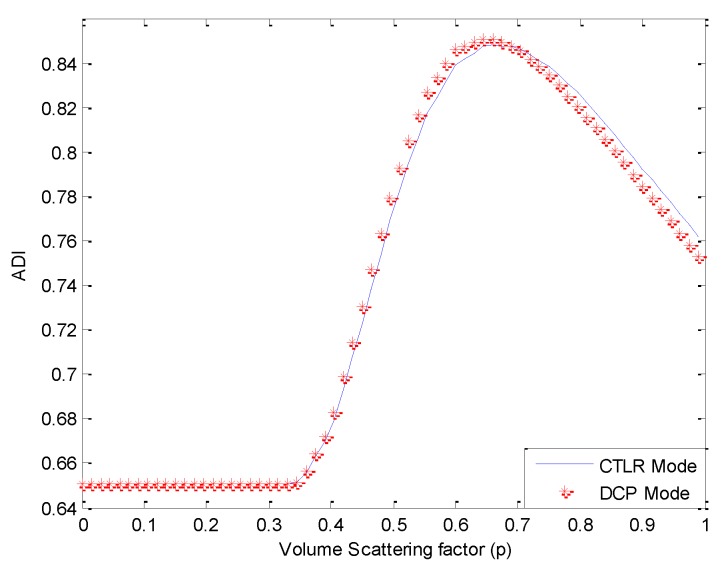
ADI under different values of *p.*

Covariance matrixes under CP modes are calculated from the FP data, and then decomposed with the proposed algorithm. We first test the decomposition performance when the value of *p* is taken as 1, as presented in Figure 9. Similar to Figure 2, the volume scattering component is overestimated in this case. Calculating ADI under different values of *p*, results are depicted in Figure 10. 

**Figure 11:**
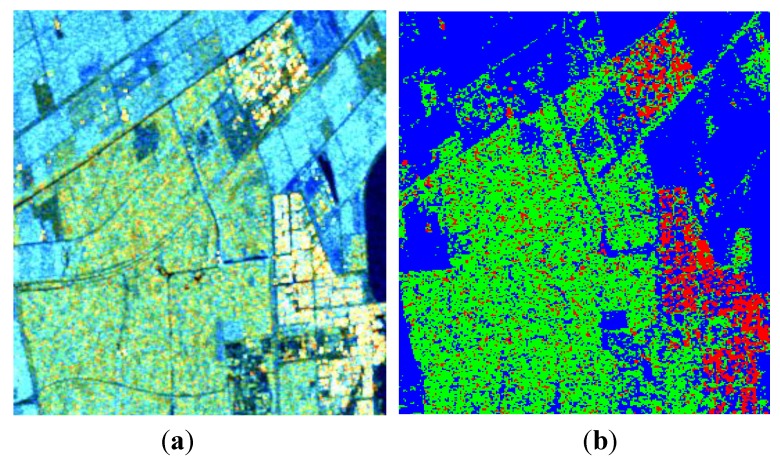
Three-component decomposition under CTLR mode when *p* = 0.65 (The original data is smoothed by a 7 × 7 pixel window): (**a**) pseudo color image and (**b**) classification.

**Figure 12:**
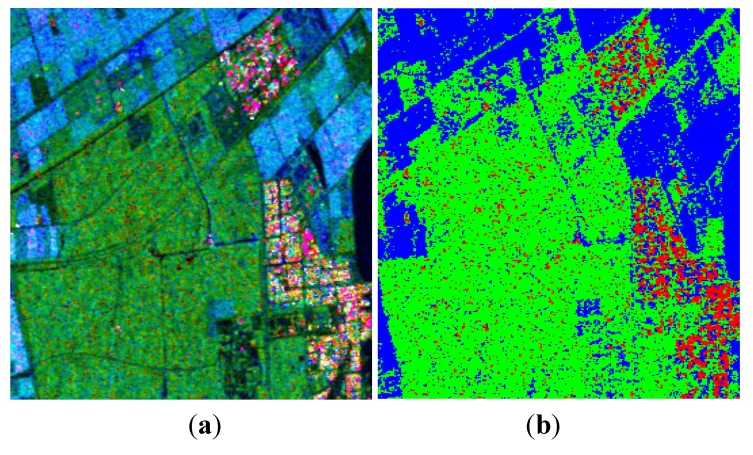
Three-component decomposition after reconstruction (the original data is smoothed by a 7 × 7 pixel window). (**a**) Pseudo color image; (**b**) Classification.

**Figure 13:**
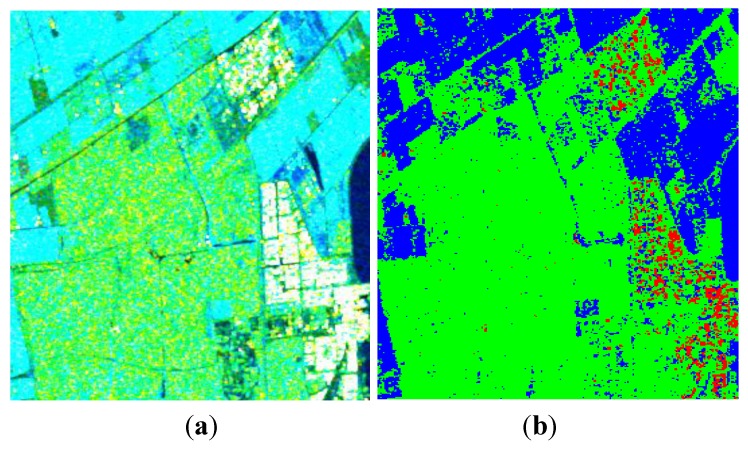
Three-component decomposition using Cloude CP decomposition (the original data is smoothed by a 7 × 7 pixel window): (**a**) pseudo color image and (**b**) classification.

**Figure 14:**
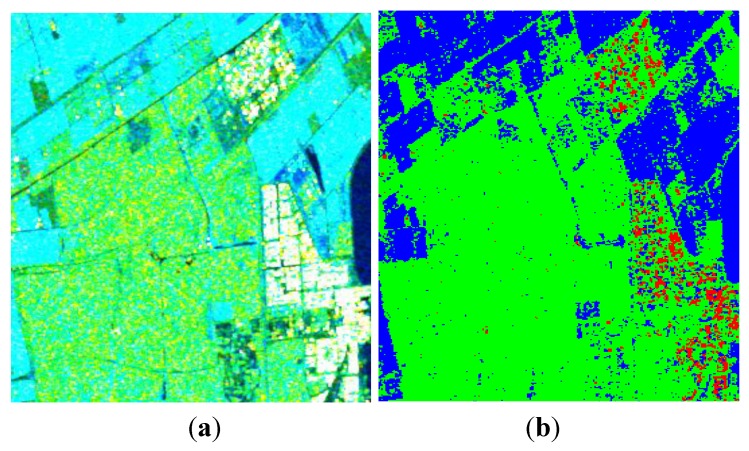
Three-component decomposition using m−δ decomposition (The original data is smoothed by a 7 × 7 pixel window). (**a**) pseudo color image and (**b**) Classification.

Similar to the last demonstration, a satisfactory ADI can be obtained when 0.5≤p≤0.8, and a maximum is reached when *p* = 0.65, as shown in Figure 11. Decomposition results after reconstruction are given in Figure 12. We also process the data with Cloude CP and m−δ decompositions, as shown in Figure 13 and Figure 14. The corresponding numerical comparisons are given in Table 5 and Table 6.

**Table 5:** Comparison by CDC and ADI.

Decomposition	CDC			ADI
	Volume	Double	Surface	
CTLR (*p* = 1)	97.26%	72.68	66.42%	75.85%
CTLR (*p* = 0.65)	74.69%	90.96%	89.68%	85.11%
CTLR(Reconstruction, us)	87.93%	72.22%	85.96%	82.04%
CTLR (Reconstruction, Nord)	83.82%	80.15%	71.49%	78.49%
CTLR (Cloude *etc.*)	98.70%	57.18%	62.31%	72.73%
CTLR (m−δ)	98.30%	66.45%	64.18%	76.31%
DCP (*p* = 1)	97.60%	71.79%	64.97%	74.94%
DCP (*p* = 0.65)	77.86%	90.51%	89.30%	85.89%
DCP(Reconstruction, us)	91.62%	71.14%	82.88%	81.88%
DCP (Reconstruction, Nord)	83.85%	84.75%	73.56%	80.72%

**Table 6:** Comparison by PCI.

Decomposition	PCI		
	Volume	Double	Surface
FP	49.79%	4.54%	45.67%
CTLR (*p* = 1)	64.56%	4.29%	31.15%
CTLR (*p* = 0.65)	41.57%	9.17%	49.26%
CTLR(Reconstruction, us)	45.58%	4.83%	49.59%
CTLR (Reconstruction, Nord)	54.76%	8.73%	36.51%
CTLR (Cloude *etc.*)	68.12%	3.06%	28.82%
CTLR(m−δ)	66.57%	3.63%	29.80%
DCP (*p* = 1)	65.41%	4.23%	30.36%
DCP (*p* = 0.65)	43.32%	8.84%	47.84%
DCP(Reconstruction, us)	48.84%	4.55%	46.61%
DCP (Reconstruction, Nord)	53.90%	8.56%	37.54%

## 8. Conclusions

This paper formulates a three-component decomposition algorithm for CTLR and DCP modes based on SV, the explicit expressions of decomposition results are derived based on setting the volume scattering component as a free parameter within a series of algebraic calculations. Different from Cloude CP and m−δ decompositions, this algorithm considers that the combined effect of double-bounce and surface and scatterings may also contribute to depolarization, thus taking the depolarized component as the upper bound of volume scattering, rather than the volume scattering component itself. 

Two typical polarimetric SAR data sets are used to demonstrate the feasibility of the proposed decomposition algorithm. If the whole depolarization is taken as volume scattering component, the performance of the proposed algorithm is similar to that of Cloude CP and m−δ decompositions. An obvious improvement can be achieved if 0.5≤p≤0.8. Since this interval is broad and decomposition performance is robust, it seems that p∈[0.5,0.8] could also be considered for other CP data. What is more, a modified $\langle {\left|S_{HV}\right|}^{2}\rangle$ reconstruction algorithm is investigated. Considering the fact that $\langle {\left|S_{HV}\right|}^{2}\rangle$ is mainly contributed by volume scattering component, the famous Souyris formula is extended to a new version. A good decomposition result could also be obtained by estimating the volume scattering component from $\langle {\left|S_{HV}\right|}^{2}\rangle$ reconstruction, with the depolarization as the upper bound.

Two studies are suggested for future work: one is to extend this algorithm to a four-component decomposition for CP SAR, and the other is to improve the accuracy of reconstruction by using different empirical formulae in different areas.
